# Workplace civility climate and its relationship with organizational citizenship behavior and intention to stay among nurses

**DOI:** 10.1186/s12912-026-05127-4

**Published:** 2026-08-11

**Authors:** Esraa Mohammed Soltan, Nahed Ahmed Hussien, Yasmine M. Osman, Nadia Mohamed El-Sayed Ghonem, Alaa Mohamed Salah El-Demerdash

**Affiliations:** 1 Department of Nursing Administration, Faculty of Nursing, Suez Canal University, 4.5 Ring Road, Ismailia, Egypt; 2 Department of Obstetric and Gynaecological Nursing, Faculty of Nursing, Suez Canal University, 4.5 Ring Road, Ismailia, Egypt; 3 Department of Obstetric and Gynaecological Nursing, Faculty of Nursing, Zagazig University, Zagazig, Egypt; 4 Department of Business Administration, Gulf College, Hafr Elbatin, Kingdom of Saudi Arabia

**Keywords:** Intention to stay, Organizational citizenship behavior, Nurses, Retention, Workplace civility climate

## Abstract

**Background:**

A workplace civility climate is a critical factor in shaping healthy nursing work environments. It influences nurses’ organizational citizenship behavior (OCB) and intention to stay, yet evidence from many healthcare systems, including Egypt, remains limited.

**Aim:**

This study aimed to evaluate the workplace civility climate and its relationship with OCB and the intention to stay among nurses.

**Methods:**

A cross-sectional descriptive correlational design was employed with 136 nurses from a hospital in Ismailia Governorate, Egypt. Data were collected using the Perceived Workplace Civility Climate Scale, the OCB Scale, and McCain’s Intent to Stay Scale.

**Results:**

Nurses reported moderate-to-high workplace civility, with the highest scores in the policies and procedures dimension. OCB also demonstrated a high overall level, particularly for altruism and courtesy. Intention to stay was moderate. Significant positive correlations were found between the total workplace civility climate and both OCB and the intention to stay.

**Conclusion:**

A positive workplace civility climate was correlated with higher OCB and greater intention to stay among nurses. Strengthening policies, reinforcing civil communication, and enhancing leadership practices may support retention and foster supportive work environments.

## Background

The nursing profession is widely recognized as the backbone of the healthcare system, directly caring for up to 90% of patients in a healthcare setting and contributing to positive healthcare outcomes [1–3]. However, the demanding nature of nursing practice often poses challenges in balancing the need for a healthy work environment with the maintenance of respectful interpersonal relationships. Consequently, enhancing supportive organizational climates has become increasingly important for nurses’ well-being, workforce retention, and quality of care [4, 5].

In this context, one key factor in cultivating a positive working environment is the workplace civility climate. The workplace civility climate is conceptualized as practices, procedures, policies, and rules that employees perceive as maintaining a minimum level of polite communication and collaboration with colleagues, supervisors, and subordinates within the professional working environment [6]. In contrast, an uncivil workplace environment encompasses disrespectful behaviors that violate workplace dignity norms, ranging from subtle belittling to overt hostility. These behaviors often lead to a reluctance to accept responsibility for errors and to intentions [7].

Civility in the workplace has been recognized as an important organizational factor associated with positive discretionary workplace behaviors among employees [8]. One important organizational outcome associated with workplace civility is organizational citizenship behavior (OCB), which refers to extra-role behaviors that support teamwork and organizational effectiveness [9, 10].

According to the Social Exchange Theory, workplace relationships are determined by reciprocity norms, whereby nurses are more likely to respond positively to favorable treatment from their organization [11]. From a social exchange perspective, such positive work experiences may be viewed as valuable social resources enabling reciprocal exchanges between nurses and their organization, and strengthening nurses’ attachment to their organization [12]. Consequently, nurses who perceive their work environment as supportive and respectful are more likely to engage in positive behaviors toward their workplace and their colleagues [13, 14]. Additionally, workplace civility is associated with nurses’ intention to stay, reflecting employees’ willingness to remain. Thus, a positive and respectful workplace can lead to better OCB and better employee retention in healthcare settings. Conversely, negative workplace environments and workplace incivility have been associated with increased intention to leave, nurse turnover, and reduced organizational commitment [15, 16].

Within the context of Egypt’s healthcare system, it is highly pluralistic and has undergone significant reforms under a recently established universal health insurance system [17]. Despite these reforms, healthcare organizations continue to face many challenges related to workforce development, supportive environments, and patient satisfaction [18]. Although workplace civility and organizational outcomes have been examined separately in previous research, there is limited evidence that the workplace civility climate simultaneously correlates with OCB and nurses’ intention to remain in Egyptian hospital settings.

Although previous studies have examined the individual effects of civility and OCB on various organizational outcomes, as well as nurses’ intention to remain in the profession, limited evidence has examined the direct relationship between workplace civility and OCB, particularly in the Egyptian healthcare setting [19, 20]. To the best of our knowledge, this study is among the first conducted in the Egyptian hospital setting to examine the joint relationships among workplace civility climate, organizational citizenship behavior, and nurses’ intention to stay.

Therefore, addressing this gap is essential not only for transforming current anecdotal and observational insights into evidence-based insights but also for developing effective strategies for a positive climate of civility. Understanding these relationships may provide important evidence for strengthening organizational climate and supporting nurse retention strategies in healthcare settings. Therefore, this study aims to examine the relationships between workplace civility, climate, OCB, and intention to stay among nurses.

## Aim

This study aims to evaluate the workplace civility climate and its relationship with OCB and the intention to stay among nurses.

### Objectives of the study

Assess nurses’ perceptions of the workplace civility climate.

Assess nurses’ perceptions of OCB.

Assess nurses’ intention to stay.

Assess the relationship between workplace civility climate and OCB among nurses.

Assess the relationship between the workplace civility climate and nurses’ intention to stay. 

### Research question

Does the workplace civility climate relate to OCB and the intention to stay among nurses?

## Methods

### Design

A cross-sectional descriptive correlational design was used.

### Setting

The study was conducted at a hospital in the north of Ismailia governorate, affiliated with the General Healthcare Authority. It has a 134-bed capacity; its scope of service includes the following: orthopaedic, pediatric, neurosurgery, spinal, and cardiothoracic surgeries.

### Participants

The participants were the nursing staff working at the selected hospital. The only exclusion criterion was having less than 6 months of experience. Except that, all nurses from different departments, age groups, and educational levels are included. The measurement of study variables doesn’t need nurses with specific criteria, except for being aware of the hospital environment. Spending six months in the hospital makes nurses aware of the hospital environment. In Egyptian governmental hospitals, all nurses’ employment status is full-time. The initial aim was to reach the calculated sample size (139 nurses) through random sampling. Still, the difficulty of data collection in the current busy hospital environment necessitated the use of convenience sampling to recruit study participants from all nursing staff at the selected hospital. Many causes lead to adopting the convenience sampling technique. These causes are the hospital’s busy working environment and the nursing staff’s heavy workload. Nursing staff resisted participating in the study, making it challenging to collect data from identified nurses. By adopting a convenience sampling technique, the researchers collect data from any nurse who agrees to participate in the study. The convenience sampling technique may limit generalizability, but in this study, all nursing categories based on age, experience, educational level, gender, residence, and marital status were represented in the sample. The sample was sufficient and representative. Adopting the convenience sampling technique was only to increase the response rate.

### Sample size

Using Epi-Info software, the minimal sample size was calculated based on an alpha of 0.05, a power (1-B) of 0.95, an effect size of 0.3, and 5 degrees of freedom. Therefore, the sample size was calculated at a 95% confidence level.

The calculated sample size was 139 nurses, and 136 agreed to participate, yielding a response rate of 97.8%.

### Instruments

Data were collected using a self-administered questionnaire composed of four parts as follows:

#### Part 1: Socio-demographic data sheet

It consists of questions about age, years of experience, educational level, marital status, gender, and residence.

#### Part 2: Perceived workplace civility climate scale

The perceived scale for assessing workplace civility was developed by Ottinot (2008) [21]. It was used in this study to assess nurses’ perception of the workplace civility climate. It includes 15 items in three dimensions: intolerance for incivility (six items), response (four items), and policies and procedures (five items).

Participants rated items using a five-point Likert scale from 1 = strongly disagree to 5 = agree. Negative items (1, 4, 6) are reverse-scored. Scale scores were calculated for each dimension, with higher scores on the response and policies/procedures dimensions indicating a higher level of perceived workplace civility climate. Items measuring intolerance for incivility were reverse-scored, with higher scores indicating that employees perceived the organization as more intolerant [21].

It demonstrates robust construct validity and high internal consistency, with Cronbach’s alpha coefficients ranging from 0.86 to 0.92, indicating excellent reliability across different organizational contexts [21]. Forward-backward translation was used to translate the scale’s English version into Arabic, ensuring its validity in the current study. First, the scale was translated into Arabic by two independent bilingual translators, who then consolidated their translations into a single clear version. This version was then back-translated into English by two separate independent translators, enabling a group of specialists to examine the text and address any inconsistencies.

The final Arabic version was subsequently reviewed by five professors of nursing administration at Egyptian nursing faculties. After the necessary modifications were made based on the nursing administration professors’ comments, a pilot test was conducted on 10% of the study sample (the participants were excluded from the study). The Arabic scale version had good content validity, and the total reliability coefficient was 0.77.

#### Part 3: OCB scale

The OCB scale was developed by Podsakoff et al. (1990) [22]. It was used to assess OCB among nurses in this study. It contains 24 items in five dimensions. Each dimension consists of 4 to 5 items. The five dimensions are conscientiousness (5 statements), altruism (5 statements), sportsmanship (5 statements), courtesy (5 statements), and civic virtue (4 statements).

The questionnaire’s validity was confirmed, and the overall scale demonstrated strong internal consistency (Cronbach’s alpha = 0.81), indicating excellent reliability [22]. Forward-backward translation was used to translate the scale’s English version into Arabic, ensuring its validity in the current study. First, the scale was translated into Arabic by two independent bilingual translators, who then consolidated their translations into a single clear version. This version was then back-translated into English by two separate independent translators, enabling a group of specialists to examine the text and address any discrepancies.

The final Arabic version was subsequently reviewed by five professors of nursing administration at Egyptian nursing faculties. After the necessary modifications were made based on the nursing administration professors’ comments, a pilot test was conducted on 10% of the study sample (the participants were excluded from the study). The Arabic scale version had robust content validity, and the total reliability coefficient was 0.89.

The scale was rated on a 5-point Likert scale, ranging from 5 (strongly agree) to 1 (strongly disagree). Negative items (6, 10) are reverse-scored. Thus, the tool’s overall score ranges from 24 to 120 [22].

#### Part 4: McCain’s intent to stay scale

McCain’s intent to stay scale was developed by McCloskey & McCain (1987) [23]. It was used to assess nurses’ intention to stay in this study. It is a 5-item scale.

The convergent construct validity of McCain’s Intent to Stay Scale was tested with McCain’s Behavioral Commitment Scale, which indicates good validity [24]. The overall Cronbach’s alpha coefficient was 0.90 [24], indicating good internal consistency. Forward-backward translation was used to translate the scale’s English version into Arabic, ensuring its validity in the current study. First, the scale was translated into Arabic by two independent bilingual translators, who then consolidated their translations into a single clear version. This version was then back-translated into English by two separate independent translators, enabling a group of specialists to examine the text and address any discrepancies.

The final Arabic version was subsequently reviewed by five professors of nursing administration at Egyptian nursing faculties. After the necessary modifications were made based on the nursing administration professors’ comments, a pilot test was conducted on 10% of the study sample (the participants were excluded from the study). The Arabic scale version had robust content validity, and the total reliability coefficient was 0.86. Responses were measured on a five-point rating scale ranging from 1 (strongly disagree) to 5 (strongly agree). The levels of the intention to stay scale are interpreted as high (score = 3.5 to 5.0), moderate (score = 2.5 to less than 3.5), and low (score = 1.0 to less than 2.5) [25].

### Procedure

The authors conducted a comprehensive literature review examining key aspects of the research problem. They visited the hospital departments at different times and contacted nurses with the assistance of the department’s head nurses. All nurses who consented to participate in the study were incorporated into the WhatsApp group. After providing participants with an overview, aim, and objectives of the study, researchers send links, including both the electronic informed consent form and questionnaires, in the WhatsApp group. Data collection was facilitated through anonymous Google Forms. To prevent duplicate responses, the Google Form setting is set to allow only one response, and the scale order was shuffled to minimize order bias. Furthermore, all questions were required to avoid missing data. Throughout the process, authors remained available to address any participant questions or concerns. Data were collected from April to June 2025.

### Data analysis

The statistical analysis was performed using SPSS system files (SPSS version 23). The normality of variables was assessed using the Kolmogorov-Smirnov test at the 0.05 level; accordingly, all main variables showed significant deviations from normality, so non-parametric tests were used. Descriptive statistics, including mean, median, standard deviation, and frequency distributions, were used to describe the variables’ characteristics. The Kruskal-Wallis test and Mann-Whitney test were used to determine the mean differences between the study sample’s demographic groups for the main study variables. The chi-square test was used to assess the relationship between nominal demographic characteristics and study variables. The Spearman correlation test was used to test the correlation between ordinal demographic characteristics and study variables. Eta-squared tests the effect size of demographic characteristics on the main study variables. The Spearman correlation test was used to answer the main study question, and multiple linear regression was used to assess the predictive relationship. Significance level values considered at *P* ≤ 0.05.

### Ethical considerations

The Research Ethics Committee at the Faculty of Nursing, Suez Canal University, approved the study proposal, assigning it approval number 305 in December 2024. The research team took ethical considerations into account regarding data confidentiality and obtained electronic informed consent from the nurses before commencing the study. We informed the participants that we would use their questionnaire responses solely for research purposes. Furthermore, their answers will not be shared with anyone outside the study team. Additionally, we applied ethical and legal principles in the current study by upholding justice and autonomy for participants. Moreover, the participants in this study had the right to withdraw at any time.

## Results

Table 1 presents the demographic characteristics of the participants. Regarding age, more than half of the participants (52.9%) were aged from 25 to less than 30 years, while 33.1% were aged from 20 to less than 25 years, and only 14.0% were aged 30 years or more. Regarding years of experience, more than half of the participants (55.8%) had fewer than 5 years, 35.3% had 5 to 9 years, and only 8.8% had 10 years or more.

Regarding educational level, the majority of the participants (59.6%) graduated from technical nursing institutes, while 30.1% held a bachelor’s degree. Smaller proportions held a nursing diploma (6.6%) or a master’s degree (3.7%). Regarding marital status, nearly two-thirds (65.4%) of participants were married, while 34.6% were single. The distribution of participants according to residence area was nearly equal, with 52.9% residing in urban areas and 47.1% in rural areas. Finally, females constituted the majority of the study sample (61.0%), compared with 39.0% males.

**Table 1:** Demographic characteristics of participants (*n* = 136)

Variables	Number	Percent
**Age (by years)**		
From 20 to less than 25	45	33.1%
From 25 to less than 30	72	52.9%
Equal to or more than 30	19	14.0%
**Experience (by years)**		
Less than 5	76	55.8%
From to 5 less than 10	48	35.3%
Equal to or more than 10	12	8.8%
**Educational Level**		
Nursing diploma	9	6.6%
Technical institute	81	59.6%
Bachelor degree	41	30.1%
Master degree	5	3.7%
**Marital status**		
Single (unmarried)	47	34.6%
Married	89	65.4%
**Residence area**		
Rural	64	47.1%
Urban	72	52.9%
**Gender**		
Female	83	61%
Male	53	39%

Table 2 presents the relationships between participants’ socio-demographic characteristics and the study variables. There were no significant relationships between gender, marital status, or residence area and any of the study variables.

**Table 2:** Relationships between participants’ socio-demographic characteristics and the study variables (*n* = 136)

Items	Workplace civility climate		OCB		Intention to stay	
	Chi square	*P* value	Chi square	*P* value	Chi square	*P* value
Gender	8.29	0.91	50.71	0.14	19.46	0.53
Marital status	42.96	0.56	99.67	0.94	31.69	0.38
Residence area	16.06	0.38	49.25	0.176	8.88	0.54

*P* ≤ 0.05

Table 3 presents the correlations between nurses’ demographic characteristics and the study variables. Years of experience showed significant weak positive correlations with workplace civility climate (*r* = 0.19, *p* = 0.03), OCB (*r* = 0.18, *p* = 0.03), and intention to stay (*r* = 0.19, *p* = 0.02). In addition, age demonstrated a significant weak positive correlation with OCB (*r* = 0.19, *p* = 0.03), whereas no significant correlations were found between age and either workplace civility climate or intention to stay. Regarding educational level, no significant correlations were observed with workplace civility climate, OCB, or intention to stay (*p* = 0.06, 0.67, and 0.07, respectively).

**Table 3:** Correlations between nurses’ demographics and the study variables (*n* = 136)

Items	Workplace civility climate		OCB		Intention to stay	
	*r*	*P* value	*r*	*P* value	*r*	*P* value
Age	0.11	0.2	0.19	0.03*	0.08	0.3
Years of experience	0.19	0.03*	0. 18	0.03*	0.19	0.02*
Educational level	-0.17	0.06	-0.04	0.67	-0.17	0.07

*r* by Spearman’s test, *P* ≤ 0.05

Table 4 demonstrates statistically significant differences among nurses with different years of experience regarding workplace civility climate, OCB, and intention to stay (*p* = 0.02, 0.03, and 0.04, respectively). The differences were reflected in the mean ranks. The mean ranks for workplace civility climate among nurses with less than 5 years of experience, from 5 to less than 10 years of experience, and 10 years or more of experience were 38.92, 70.32, and 73.01, respectively. Similarly, the mean ranks for OCB among nurses with less than 5 years of experience, from 5 to less than 10 years of experience, and 10 years or more of experience were 59.45, 71.08, and 75.84, respectively. Regarding intention to stay, the mean ranks were 63.02, 71.89, and 89.67, respectively.

Furthermore, a significant difference in OCB was observed across age groups (*p* = 0.02). The mean ranks of nurses aged from 20 to less than 25 years, from 25 to less than 30 years, and 30 years or older were 50.43, 70.68, and 72.97, respectively. However, no significant differences were found in educational level, marital status, residence area, or gender regarding workplace civility climate, OCB, or intention to stay.

**Table 4:** Mean differences between nurses’ socio-demographic characteristics and workplace civility climate, OCB, and intention to stay (*n* = 136)

Variables	Workplace civility climate			OCB			Intention to stay		
	Mean rank	Chi –square	*P* value	Mean rank	Chi –square	*P* value	Mean rank	Chi -square	*P* value
**Age (by years)**									
From 20 to less than 25	67.36	3.23	0.20	50.43	2.87	0.02*	65.61	1.96	0.38
From 25 to less than 30	72.84			70.68			67.26		
Equal to or more than 30	54.76			72.97			80.05		
**Experience (by years)**									
Less than 5	38.92	7.58	0.02*	59.45	2.97	0.03*	63.02	5.33	0.04*
From to 5 less than 10	70.32			71.08			71.89		
Equal to or more than 10	73.01			75.84			89.67		
**Educational Level**									
Nursing diploma	50.33	2.92	0.40	61.11	0.90	0.83	71.17	4.61	0.20
Technical institute	70.33			68.67			73.29		
Bachelor degree	70.67			68.15			61.52		
Master degree	53.8			43.3			43.3		
**Marital status**									
Single (unmarried)	66.8	0.37	0.71	65.07	0.74	0.46	69.5	2.38	0.08
Married	69.4			70.31			74.31		
**Residence area**									
Rural	65.32	0.89	0.37	66.86	0.46	0.65	64.16	1.31	0.22
Urban	71.33			69.96			72.35		
**Gender**									
Female	67.63	0.32	0.75	69.65	1.93	0.07	70.19	0.63	0.53
Male	69.86			63.30			65.85		

*P* ≤ 0.05 Z by Mann-Whitney Chi-Square by Kruskal-Wallis

Table 5 demonstrates a small but significant effect of years of experience on workplace civility climate, OCB, and intention to stay (*p* = 0.03, 0.01, and 0.04, respectively). The effect sizes were small, with η² values of 0.038, 0.018, and 0.039, respectively. Additionally, age had a small, statistically significant effect on OCB (*p* = 0.04, η² = 0.013). However, no significant effects were found for educational level, gender, marital status, or residence on workplace civility climate, OCB, or intention to stay.

**Table 5:** The effect size of demographic characteristics on study variables (workplace civility climate, OCB, and intention to stay) (*n* = 136)

Variables	Workplace civility climate		OCB		Intention to stay	
	*P* value	η 2	*P* value	η 2	*P* value	η 2
Age	0.42	0.013	0.04*	0.013	0.81	0.012
Experience	0.03*	0.038	0.01*	0.018	0.04*	0.039
Educational level	0.53	0.017	0.66	0.012	0.07	0.061
Gender	0.95	0.001	0.13	0.017	0.29	0.008
Marital status	0.65	0.002	0.86	0.001	0.39	0.031
Residence	0.29	0.008	0.58	0.002	0.28	0.009

*P* ≤ 0.05

Table 6 presents the descriptive statistics of workplace civility climate as perceived by nurses. The total mean score for workplace civility climate was 3.47 ± 0.61. Among the individual dimensions, policies and procedures had the highest mean score (3.62 ± 0.98). This was followed by intolerance for incivility, with a mean score of 3.49 ± 0.81. In contrast, the response dimension had the lowest mean score (3.29 ± 0.76).

**Table 6:** Descriptive statistics of workplace civility climate as perceived by nurses (*n* = 136)

Items	Mean ± SD	Median	Minimum	Maximum
Intolerance for incivility	3.49 **±** 0.81	3.5	1.67	5
Response	3.29 **±** 0.76	3.2	1	5
Policies and procedures	3.62 **±** 0.98	3.75	1	5
Total workplace civility climate	3.47 **±** 0.61	3.47	1.77	5

Table 7 presents the descriptive statistics of OCB among nurses. The total mean OCB score was 96.71 ± 11.53. Regarding the individual dimensions, the highest mean scores were observed for altruism (21.23 ± 3.27) and courtesy (21.14 ± 3.23). Additionally, conscientiousness and sportsmanship demonstrated relatively high mean scores of 19.57 ± 2.82 and 19.42 ± 3.81, respectively. In contrast, civic virtue had the lowest mean score (15.36 ± 2.88).

**Table 7:** Descriptive statistics of OCB among nurses (*n* = 136)

Items	Mean ± SD	Median	Minimum	Maximum
Altruism	21.23 ± 3.27	21	13	25
Sportsmanship	19.42 ± 3.81	19.5	5	25
Conscientiousness	19.57 ± 2.82	20	13	25
Courtesy	21.14 ± 3.23	20	13	25
Civic virtue	15.36 ± 2.88	16	7	20
Total OCB	96.71 ± 11.53	97	72	120

Table 8 presents the descriptive statistics for nurses’ intention to stay. The total mean score for intention to stay was 3.49 ± 0.83. Among the individual items, the highest mean score (3.66 ± 0.96) was reported for the statement, “I would recommend this organization as a good place to work to others,” with nearly two-thirds of the participants (64.7%) expressing agreement. Similarly, high levels of agreement were observed for items related to continued career commitment within the organization (61.0%, mean = 3.63 ± 1.02) and plans to remain with the organization for the foreseeable future (55.9%, mean = 3.57 ± 0.96). In contrast, the lowest mean score (3.10 ± 1.08) was recorded for the statement, “I am unlikely to look for a job outside of this organization within the next year.” Responses to this item were more variable, with the largest proportion of participants (39.0%) selecting the neutral response.

**Table 8:** Descriptive statistics of intention to stay among nurses (*n* = 136)

Item	Agree		Neutral		Disagree		Mean ± SD
	*N*	%	*N*	%	*N*	%	
1-I plan to remain with this organization for the foreseeable future	76	55.9%	47	34.6%	13	9.6%	3.57 ± 0.96
2-I see a long-term future for myself in this organization	72	52.9%	49	36%	15	11%	3.49 ± 0.94
3-I would recommend this organization as a good place to work to others	88	64.7%	33	24.3%	15	11%	3.66 ± 0.96
4-I am unlikely to look for a job outside of this organization within the next year	46	33.8%	53	39%	37	27.2%	3.10 ± 1.08
5-I feel committed to continuing my career with this organization	83	61%	38	27.9%	15	11%	3.63 ± 1.02
Total intention to stay							**3.49 ± 0.83**

**Fig. 1:**
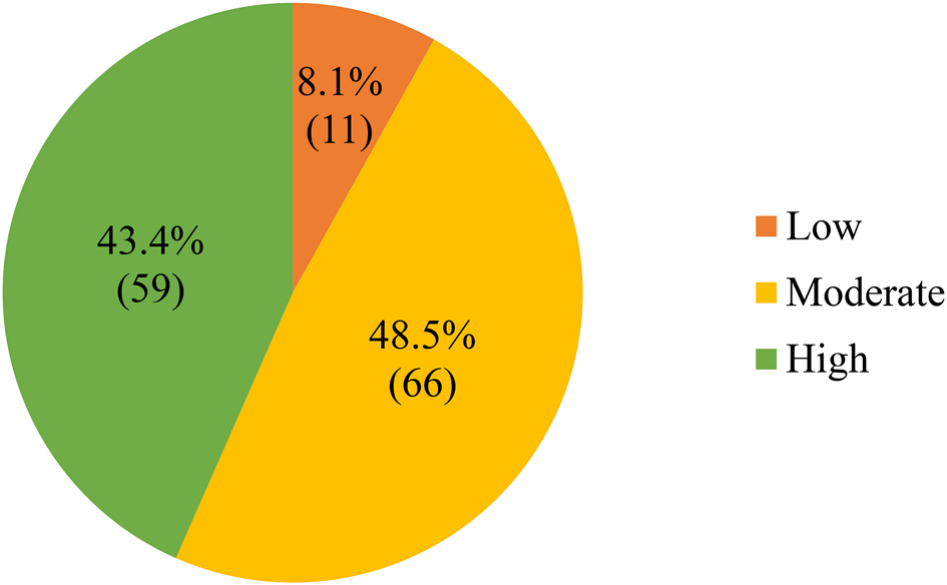
Intention to stay levels among nurses (*n* = 136)

Figure 1 illustrates the distribution of intention-to-stay levels among nurses: nearly half (48.5%) demonstrated a moderate level, while a considerable proportion (43.4%) reported a high level. In contrast, only a small minority of nurses (8.1%) had a low intention to stay.

Table 9 demonstrates the correlations between workplace civility climate, OCB, and the intention to stay among nurses. A significant moderate positive correlation was found between the total workplace civility climate score and total intention to stay (*r* = 0.317, *p* = 0.001). In addition, a significant weak positive correlation was observed between the total workplace civility climate score and total OCB (*r* = 0.255, *p* = 0.003). Among the dimensions of workplace civility, policies and procedures showed the strongest and most consistent relationships with OCB dimensions and intention to stay. Specifically, it demonstrated moderate positive correlations with civic virtue (*r* = 0.456, *p* = 0.001), sportsmanship (*r* = 0.339, *p* = 0.001), altruism (*r* = 0.347, *p* = 0.001), and total intention to stay (*r* = 0.359, *p* = 0.001). Furthermore, altruism, sportsmanship, courtesy, and civic virtue exhibited weak to moderate positive correlations with several dimensions of workplace civility climate. In contrast, conscientiousness was not significantly correlated with any dimension of the workplace civility climate (*p* > 0.05).

**Table 9:** Correlation between workplace civility climate, OCB, and the intention to stay among nurses (*n* = 136)

Items	Intolerance for incivility		Response		Policies and procedures		Total workplace civility climate	
	*R*	*P* value	*r*	*P* value	*r*	*P* value	*R*	*P* value
Altruism	0.184	0.032*	0.196	0.022*	0.347	0.001*	0.346	0.001*
Sportsmanship	0.437	0.001*	-0.081	0.35	0.339	0.001*	0.414	0.001*
Conscientiousness	0.014	0.869	0.101	0.240	0.059	0.493	0.077	0.372
Courtesy	0.231	0.007*	0.162	0.059	0.183	0.033*	0.267	0.002*
Civic virtue	0.201	0.019*	0.254	0.003*	0.456	0.001*	0.421	0.001*
Total OCB	0.062	0.471	0.247	0.004*	0.263	0.002*	0.255	0.003*
Total intention to stay	0.112	0.193	0.229	0.007*	0.359	0.001*	0.317	0.001*

Table 10 presents the results of multiple linear regression analysis examining the effects of OCB and intention to stay on workplace civility climate. The regression model was significant (F = 9.37, *p* = 0.001) and explained 12.4% of the variance in workplace civility climate (R² = 0.124). Both OCB (B = 0.211, *p* = 0.04) and intention to stay (B = 0.165, *p* = 0.002) were significant positive predictors of workplace civility climate. Additionally, no multicollinearity was detected among the predictor variables (VIF = 1.089).

**Table 10:** Multiple linear regression between OCB, intention to stay, and the workplace civility climate (*n* = 136)

Dependent variables	Predictor variables	*R*	*R* square	F	F significance	B	t	t significance	VIF
Workplace civility climate	**OCB**	0.351	0.124	9.37	0.001	0.211	1.897	0.04*	1.089
	**Intention to stay**					0.165	3.184	0.002*	

## Discussion

The present study found no significant differences in workplace civility climate, OCB, and intention to stay across nurses’ demographic characteristics. This overarching finding contradicts the findings of a prior study conducted in Saudi Arabia [26]. While some literature supports specific null findings, researchers have found no significant differences in OCB and workplace civility climate based on gender or educational level [27–29]. The current study’s broad, non-significant results stand in contrast to those of a South Korean study [30], which reported significant differences in intention to stay based on marital status and educational level. These findings suggest that localized institutional experiences, rather than fixed demographic identities, shape organizational climate, voluntary behaviors, and retention choices. 

Several significant associations emerged between nurses’ demographic characteristics and the study variables. Years of experience demonstrated a weak positive association with the workplace civility climate, OCB, and nurses’ intention to stay, aligning with the previous studies on OCB [26] and nurses’ intention to stay [31]. This trend can be attributed to the professional maturity of seasoned nurses, who possess the systemic familiarity required to navigate organizational stressors, engage in discretionary helping behaviors, and maintain higher institutional loyalty.

The present study revealed that age significantly correlated with OCB, contradicting the results reported by other researchers [26]. Conversely, age showed no significant correlation with either workplace civility or the intention to stay. These results diverge from some literature on workplace civility climate [32] and intention to stay [25]. However, the observed effect sizes were small, indicating that while experience contributes to these outcomes, it does not account for substantial variance. This reinforces the conclusion that workplace factors remain more influential than demographic characteristics.

The total mean score for the workplace civility climate in the current study was moderate to high, indicating a strong overall perception of civility among nurses. This contrasts with the lower perception reported in a 2024 study [33]. Concerning the dimensions of workplace civility climate, policies and procedures received the highest mean score, suggesting that nurses recognize the formal structures and rules designed to promote civility. On the other hand, the lowest score was reported for the dimension of response, indicating a perceived gap between policy formulations and their effective implementation or follow-up when incivility occurs. While intolerance for incivility was rated as average. These findings are consistent with two studies [34, 35] but contrast with Hossny & Sabra (2020) [32], highlighting the need for targeted organizational improvement in the practical application of civility measures.

The hypothesized link between the low civic virtue and the low response dimension scores suggests that nurses may withdraw from organizational loyalty/participation due to limited trust in management’s ability to handle incivility, while still maintaining positive peer-to-peer behaviors.

The current study revealed a high total level of OCB among the nurses. This finding aligns with other studies conducted in Egypt [36, 37]; however, it contradicts findings on the relationship between nurses’ OCB and their job attitudes [38]. All five OCB dimensions also demonstrated high mean scores, indicating the nurses’ priorities in OCB. The highest mean scores were recorded for altruism and courtesy, which involve direct interpersonal helpfulness and politeness/preventive problem-solving, respectively. The mean score for civic virtue, which shows how involved people are in the organization, was the lowest, but it was still high. This hierarchy is consistent with the findings of an Egyptian study [36] but differs from those reported by other researchers [39, 40]. Thus, the observed hierarchy of OCB dimensions supports the interpretation that nurses prioritize immediate interpersonal responsibilities over broader organizational involvement, particularly where participatory structures may be limited.

The total mean score for nurses’ intention to stay in the present study indicates a moderate level. This finding suggests that, while nurses are not actively seeking immediate departure, their commitment remains tentative, consistent with findings from Saudi Arabian and Jordanian studies [16, 41] in similar healthcare contexts. Regarding intention to stay items, the strongest consensus was to recommend the organization as a good place to work, indicating a positive perception of the hospital’s reputation, culture, and quality of care. Strong agreement was also found for maintaining career commitment and planning to remain for the foreseeable future. In contrast, the lowest mean score was on “I am unlikely to look for a job outside of this organization within the next year.” This finding highlights a significant tension: nurses generally value the organization but remain open to external job opportunities, underscoring the proactive retention strategies, aligning with the literature linking a negative work environment to higher intentions to leave [42].

The current study results indicate that workplace civility climate is a significant factor in OCB and retention. A moderate positive correlation was found between the total civility climate score and total intention to stay, suggesting that a better civility climate is associated with a greater likelihood that employees will remain with the organization. This result aligns with prior studies linking civility or caring climates to lower turnover intention [35, 43, 44]. In contrast, the relationship between the total civility climate and OCB was a weak positive correlation, which implies that a more civil environment is associated with a slight increase in OCB. This finding is consistent with two studies published in 2020 and 2024, which reported a positive relationship between the workplace civility climate and OCB [45, 46].

The total workplace civility climate showed a low correlation with OCB but a high correlation with intention to stay, as indicated by the difference in correlation strengths. Because OCB is highly variable and influenced by many factors, a civil environment function is a fundamental requirement for organizational justice, which significantly influences the decision to stay, making it a stronger driver of retention than voluntary behavior.

Among the dimensions of workplace civility climate, policies and procedures showed the strongest and most consistent positive relationships, correlating moderately with civic virtue, sportsmanship, altruism, and intention to stay. This indicates that clear and fair organizational rules are significantly associated with a more civil workplace, greater engagement in positive workplace behavior, and higher employees’ intention to stay, supporting the idea presented by Abugre (2017) [47]. Finally, the remaining OCB dimensions correlated weakly to moderately with other workplace civility climate dimensions, reinforcing the finding that improving the civility climate generally increases OCB, though other factors also contribute.

The regression model indicates that both OCB and intention to stay significantly predict workplace civility climate, although the explained variance was modest. This suggests that civility climate is both an outcome and a reinforcing mechanism within organizational dynamics. However, the relatively modest explained variance by OCB and intent to stay indicates that other organizational and individual factors contribute to workplace civility climate. Recent literature conceptualizes workplace climate as a reciprocal system in which employee behaviors (e.g., OCB) and attitudes (e.g., intention to stay) both influence and are influenced by the organizational environment [48]. The relatively modest explanatory power suggests the presence of additional unmeasured variables, such as leadership style, workload, and psychological well-being, which have been consistently identified as key determinants in recent research [49].

### Limitations

Despite the valuable insights gained, this study has several limitations that should be acknowledged. First, the cross-sectional correlational design prevents the causal pathways and conclusions. While significant associations were identified, it remains unclear whether high-performing teams perceive their climate more favorably. Second, due to the busy hospital environment and heavy nurse workloads, the researchers shifted from random to convenience sampling. Relying on convenience sampling within a single hospital limits the generalizability of our findings. It may introduce selection bias, since nurses with more favorable views or greater availability were more likely to participate, which potentially inflates the reported OCB levels.

Third, since organizational culture varies across Egyptian healthcare authorities and regions, findings from one hospital in Ismailia Governorate may not represent the broader nursing population. Finally, data were self-reported via electronic questionnaires. This method is susceptible to social desirability bias, in which participants may over-report positive behaviors, such as altruism and conscientiousness, or under-report negative perceptions of incivility.

## Conclusion

Based on this study’s findings, it can be concluded that nurses reported a moderate-to-high level of perceived workplace civility, with policies and procedures as the strongest component. OCB was also high across all its dimensions. Additionally, most nurses reported moderate to high intentions to stay. Significant positive correlations were found between workplace civility climate and both OCB and intention to stay. 

However, given the study’s cross-sectional design, these relationships should be interpreted as associative and do not imply causal effects.

### Recommendations

Based on the study findings, the following evidence-based recommendations are proposed to enhance the work environment and support nurse retention:

### For practice

- Strengthen the response dimension through actionable enforcement mechanisms.
- Target leadership training specifically to address gaps in response and enforcement.
- Leverage strong policies and procedures to enhance retention strategies.
- Develop interventions to increase trust in managerial responses and improve the intention to stay.
- Enhance civic engagement through participatory structures.
- Align civility initiatives with interpersonal strengths.

### For research

- Further studies are recommended to explore causal pathways between civility response, OCB, and intention to stay.
- Future research should expand the scope to multi-center, probability-based studies to address the generalizability limitations of single-hospital convenience sampling.

## Data Availability

The data will be made available from the corresponding author upon reasonable request.

## Abbreviations

OCB: Organizational Citizenship Behavior
